# Study on the Impact of Laser Settings on Parameters of Induced Graphene Layers Constituting the Antenna of UHF RFID_LIG_ Transponders

**DOI:** 10.3390/s25061906

**Published:** 2025-03-19

**Authors:** Aleksandr Kolomijec, Piotr Jankowski-Mihułowicz, Mariusz Węglarski, Nikita Bailiuk

**Affiliations:** 1 Doctoral School, Rzeszów University of Technology, al. Powstańców Warszawy 12, 35-959 Rzeszów, Poland; 2 Department of Electronic and Telecommunications Systems, Rzeszów University of Technology, ul. Wincentego Pola 2, 35-959 Rzeszów, Poland; wmar@prz.edu.pl (M.W.); n.bailiuk@prz.edu.pl (N.B.)

**Keywords:** RFID, laser-induced graphene, RFIDtex tag, RFID_LIG_ transponder, laser, smart textiles, graphene antenna, UHF band, impedance matching

## Abstract

The aim of the research is to investigate the impact of laser operation parameters on the LIG (laser-induced graphene) process. It focuses on evaluating the feasibility of using the induced conductive layers to create antenna circuits that are dedicated to radio-frequency identification (RFID) technology. Given the specific design of textile RFIDtex transponders, applying the LIG technique to fabricate antenna modules on a flexible substrate (e.g., Kapton) opens new possibilities for integrating RFID labels with modern materials and products. The paper analyses the efficiency of energy and data transmission in the proposed innovative UHF RFID_LIG_ tags. The signal strength, read range, and effectiveness are estimated in the experimental setup, providing key insights into the performance of the devices. Based on the obtained results, it can be concluded that changes in laser cutting parameters, the size of the induced graphene layer, and the method of fixing the Kapton substrate significantly affect the quality of the cutting/engraving components and the conductivity of burned paths. However, these changes do not directly affect the correct operation of the RFID_LIG_ transponders, owing to the fact that these structures are resistant to external impacts. Nevertheless, an increased range of data readout from the RFID_LIG_ tags can be achieved by using graphene paths with higher conductivity. The obtained results confirm the validity of the proposed concept and provide a foundation for further research on adapting the LIG method to automated logistics, ultimately leading to the development of more versatile and innovative solutions for identification processes.

## 1. Introduction

### 1.1. Aim of Research

The discovery of graphene in 2004 initiated numerous scientific studies on this carbon allotrope. Due to its exceptional mechanical, chemical, optical, and electrical properties, the scope of its applications has been continually expanding [1,2]. Alongside this, new methods of obtaining graphene are being sought, and existing methods are being improved [3]. In 2014, James M. Tour’s team synthesized graphene using a laser beam [4]. The method of laser-induced graphene (LIG) is also referred to as laser ablation graphene (LAG), laser-scribed graphene (LSG), or laser-derived graphene (LDG). Typically, in the LIG method, graphene is produced using a CO_2_ infrared laser with a light emission wavelength of 10.6 μm [5,6]. With the advancement of laser systems, much more precise devices have emerged. UV lasers, with wavelengths typically in the range of 355–266 nm, offer higher precision and finer structural details [7,8]. Diode lasers operating across a broad range, from visible light to near-infrared (405–1550 nm), are compact, energy-efficient, and versatile, making them suitable for a wide range of technologies. Nd:YAG lasers, emitting primarily at 1064 nm, are also used due to their high power and ability to generate precise, high-quality graphene structures.

Laser beam generates locally high temperature (exceeding 2500 °C), causing the breaking of N-C and C-O bonds in the processed material, the rearrangement of carbon atoms, and the formation of porous 3D graphene structures on substrate surfaces [5,6]. In this way, graphene can be induced on any material containing carbon, such as wood, food, clothing, paper, polyimide (PI) [7], and others. The simplicity of this synthesis process and the relatively high electrical conductivity of the resulting graphene structure leads to a growing interest in the LIG method within the electronic industry. This increasing focus also reflects in the manufacturing processes of circuits designed for radio-frequency identification (RFID) devices.

Many research centers conduct numerous studies on adapting the laser-induced graphene method for fabricating various types of antenna system components [9,10,11]. Theoretical considerations and experimental efforts aimed at evaluating the advantages and disadvantages of this method compared to other modern electronic circuit technologies are also carried out [12]. These investigations focus on optimizing the performance of radio frequency front-ends by improving signal quality, efficiency, and miniaturization, while addressing challenges related to material properties and scalability, including modifications in the shapes of designed graphene layers [11] or implementations of unconventional techniques for inducing carbon allotropes [9,12].

Considering the existing literature, the authors aimed to improve the efficiency of the antenna front-end by modifying the settings of the laser device while maintaining the shape of the induced graphene components. The laser parameters were selected to ensure that the resulting layers exhibited optimal properties, making them suitable for use in RFID systems of the ultra-high frequency (UHF) band.

### 1.2. Textronic Transponder RFIDtex Without Galvanic Coupling

In numerous scientific studies as well as engineering projects, the problem of integrating RFID transponders with everyday objects frequently arises, with particular attention recently given to e-textiles [13]. In mass textile production, the use of traditional RFID tags made from aluminum, copper, or other metals is a subject of debate [12,14]. Although their performance is the best among the currently known options, and they are manufactured using well-established technological methods, environmental concerns regarding the consequences of the large-scale fabrication of metal antennas, as well as the need to minimize the prolonged direct contact of the human body with such antennas, make it necessary to develop new designs.

This issue is even more significant considering that one of the European Union’s strategies is to introduce the so-called Digital Product Passport. This solution may, in the future, be based on RFID systems. The policy aims to provide consumers with comprehensive information about purchased products, including their material composition, manufacturing processes, environmental impact, and disposal methods. According to the Circular Economy Action Plan, these principles are set to apply starting in 2026 to industries with the greatest environmental impact, such as battery production, electronics manufacturing (including household appliances), and, crucially, textiles—which are directly relevant to the R&D efforts described in this paper.

A further extensive range of additional implementation possibilities opens up when RFID transponders are equipped with sensors for various physical quantities. Examples include tags that monitor human health [15,16,17], activities of workers, officers or the elderly activity, as well as location [18,19], etc. These applications primarily leverage solutions from flexible electronic technologies. One of the emerging challenges is the use of conductive threads to create pathways in electronic circuits [20,21]. Research on adapting conductive threads for the fabrication of RFID antennas [22,23] demonstrates their applicability and potential. However, according to [24,25], a significant disadvantage of conductive threads is the degradation of conductivity due to the separation of silver microparticles during use, especially in washing processes.

The concept of the RFIDtex transponder-sensor [26] is an ideal approach to address the problem of integrating RFID functionality into everyday textile products. It is specially designed to leverage the unique properties of conductive threads in order to achieve a full integration of the antenna with textile substrates, such as garments. Interestingly, graphene-based pathways can also serve as a substitute for conductive threads, enabling the creation of antennas on a broad range of materials containing carbon. Additionally, the specific design of the RFIDtex tag simplifies the task of integrating the antenna with the semiconductor integrated circuits (ICs), as there is no need to use any soldering or adhesive bonding technologies.

Therefore, the conducted research aims to explore the feasibility of using the LIG method [11,27] in such applications. It is important to emphasize that, due to the fragility of the graphene layers, direct soldering of the ICs to the electronic circuitry is not feasible. Hence, new methods for connecting the graphene-based layer are being explored. For example, in [27], silver ink is used to bond with the LIG paths, forming a conductive LIG/Ag composite. In [11], the graphene loop is inductively coupled with a smaller rectangular loop.

The proposed design of the UHF RFID_LIG_ transponder, in which the antenna is fabricated as a thin and delicate graphene layer, is elaborated as an extension of the concept of the textronic RFIDtex sensor. The concept and operation of the RFIDtex transponders are described in a previous publication by the authors [26].

**Figure 1:**
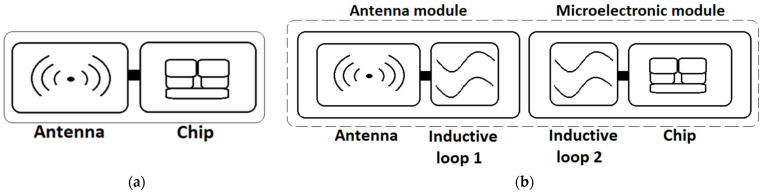
Block diagram of UHF RFID transponder (tag): (**a**) typical passive construction; (**b**) RFIDtex design with separate antenna module and microelectronic module.

In the classic version of an RFID transponder, the chip (IC) is galvanically connected to the antenna, forming a unified electronic structure (Figure 1a). This connection is typically achieved through bonding, soldering, or gluing. While this type of construction may not pose significant challenges for production lines, it is particularly susceptible to environmental factors, especially when the antennas are fabricated using unconventional means, such as conductive threads or LIG layers. It is important to emphasize that any inaccuracies in the RF front-end of the tag can lead to an impedance mismatch at the input terminals of the control chip and it significantly impacts the overall efficiency of the entire RFID system. In contrast, the patented RFIDtex construction [26] that is specifically designed for integration with textile products, consists of the antenna module and the chip module that are galvanically separated components, fabricated on various substrates, in diverse technologies. Their connection is achieved through the coupling of two inductive loops (constitute coupling system) (Figure 1b). The antenna, together with the first coupling circuit, forms the antenna module. The chip, along with the second coupling circuit, constitutes the microelectronic module.

Galvanic separation between both the modules enables the use of different production methods for each module, while ensuring seamless integration into a unified system. For example, the antenna module can be fabricated by sewing with conductive threads, while the microelectronic part can take the form of an electronic semiproduct, such as a button, which can be attached to the fabric using standard techniques commonly employed in the textile industry [26]. Numerous studies on systems utilizing inductive coupling for component integration, conducted by the authors [13,26,27,28], confirm the reliability of the results obtained with this method.

### 1.3. Transponder RFID_LIG_ with Laser-Induced Graphene

Due to the fragility of the induced graphene, there is the need to eliminate the galvanic connection with any IC chips. Thus, the RFID_LIG_ transponder consists of the antenna module separated from the microelectronic module. The antenna is made up of a LIG path, whereas the coupling function is performed by the central part of the path, which overlaps with the coupling loop of the microelectronic module. For the microelectronic module, the near-field tags from Talkin’Things (designated as TT) are utilized. Additionally, the authors’ own design 5.7x20_v001, was also specially developed for the needs of this study. The used constructions of the microelectronic modules are discussed in more detail in Section 2.2.

In the study, the graphene layer of the RFID_LIG_ transponder was fabricated in the shape of a dipole antenna, which is one of the typical designs used for antennas of the UHF band. Other common constructions include monopole, microstrip antennas, etc., [29,30]. Attempts were made to induce graphene in the two configurations of the dipole: a standard straight path and a dipole with a loop coupling circuit in the center, as described in publications [13,30]. After the initial unsuccessful efforts to fabricate the second version, the focus was shifted to the simpler construction. The reason for this decision and associated issues are discussed in Section 3.1.

## 2. Materials and Methods

### 2.1. LIG Technology

The test samples were prepared in Inkspace v.1.2.2 and TROTEC JobControl v.10.7.1 software tools and fabricated using the Trotec FineMaker Hybrid Strong Laser (Trotec Laser Poland, Warsaw, Poland). The graphene layers were induced on a Kapton substrate. First, the shape of the induced antenna was designed in Inkspace. Then, the project was converted into a format compatible with the TROTEC JobControl software.

The TROTEC JobControl tool facilitates precise management of laser-based processes, including engraving and cutting, by providing the user-friendly interface to adjust main technological parameters. It enables users to optimize settings, such as laser power, speed, and beam emission frequency, ensuring that the process can be tailored to the specific requirements of the chosen materials. In the presented work, both the “Cut CO_2_” and “Engrave” operating modes were investigated. When switching between these modes, one of the primary adjustable parameters changes as well. In “Cut CO_2_”, the pulse frequency *f_ran_* emitted by the laser head can be set within the range of 1 kHz to 60 kHz. In “Engrave”, the editable parameter is *PPI_ran_* (pulses per inch), which defines the number of laser pulses emitted per inch of length, with a range of 500 to 1000 impulses. Regardless of the head movement speed, these pulses are always emitted at regular time intervals. In TROTEC JobControl tool, the power and speed parameters for the laser beam movement are set as a percentage of the maximum achievable value [31]. For the FineMarker Hybrid Strong system, *P_max_* = 45 W [32], *v_max_* = 3.5 m/s [33].

The theoretical width of the induced graphene path was edited in Inkspace tool. However, the final width is determined by the height of the laser head above the material surface. The OZ coordinate defines the laser’s focal point and can be also adjusted in TROTEC JobControl software. Based on the geometrical parameters defined in the user’s design, the laser system automatically adjusts the cutting process, which can be either one-way or two-way cutting. In the first case, the laser beam moves from the left end to the right, continuously processing the entire modeled shape, and the laser head remains constantly active for components with full coverage. In two-way mode, the laser beam passes halfway along the defined path, starting from one side of the shape, and then switches off at the midpoint, repositions to the opposite end, and repeats the entire process in a mirrored image. During the experiment, the method of fixing the Kapton and adjusting the laser cutting parameters were optimized to achieve the path with the highest electrical conductivity. The resistance of individual paths was measured using a multimeter by placing its probes at the ends of the graphene path.

All test samples were fabricated on a single Kapton sheet. To create functional RFID_LIG_ transponders, the sheet was cut into individual strips, each containing a single LIG path serving as antenna. In the subsequent part of the experiment, seventeen test samples were evaluated. A summary of the laser settings and the resistance measurements of individual samples is provided in Section 3.1.

### 2.2. Evaluation of RFID_LIG_ Transponders

**Figure 2:**
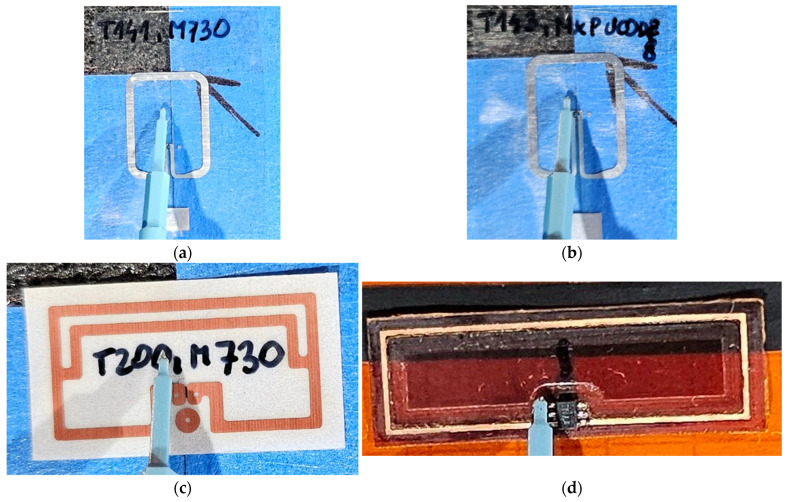
Microelectronic modules: (**a**) TT-141; (**b**) TT-143; (**c**) TT-200; (**d**) 5.7 × 20_v001.

According to the concept of the RFID_LIG_ transponder, which follows the construction principles of the textronics RFIDtex tags, each sample with induced graphene forms the antenna module inductively coupled with the microelectronic module. The microelectronic modules used in the study were the TT-141, TT-143, and TT-200 near-field tags from the Talkin’Things portfolio [34], as well as the 5.7x20_v001 design, created according to the authors’ own concept (Figure 2).

It should be noted that the TT components were originally developed as ready-made UHF RFID tags, commercial products designed for operating in very close proximity to the read/write device (RWD). According to the Talkin’Things’ intentions, these transponders were not intended for use in the RFIDtex or RFID_LIG_ devices. However, they were repurposed as microelectronic modules for the present investigations, which aimed to validate the proposed assumptions. In contrast, the module designed by the authors is an electronic circuit especially elaborated for the conducted experiments, and it cannot operate as a standalone transponder.

**Figure 3:**
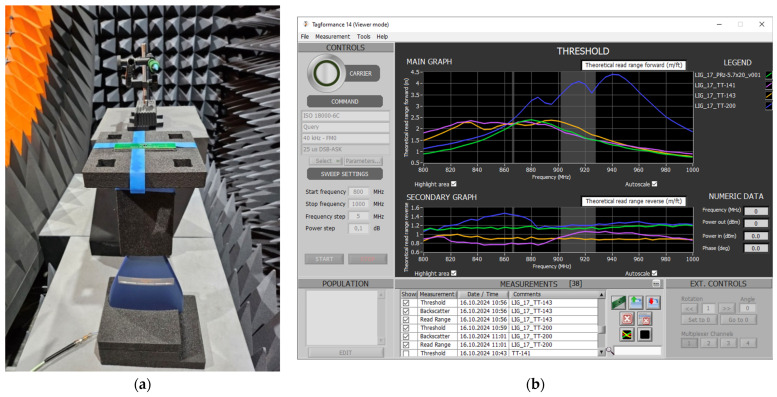
Laboratory stand for measuring read range of RFID_LIG_ transponders: (**a**) stand in anechoic chamber; (**b**) Voyantic Tagformance Pro software tool.

The read range parameter of the designed RFID_LIG_ transponders were measured in the anechoic chamber of Microwave Vision Group (Figure 3a) on the stage equipped with the Voyantic Tagformance Pro system (Voyantic, Helsinki, Finland) and Tagformance UHF v.14 (Figure 3b).

Before starting the measurement, the parameters of the communication protocol ISO/IEC 18000-63 were configured according to the EPC Class 1 Gen 2 [35] (Table 1).

**Figure 4:**
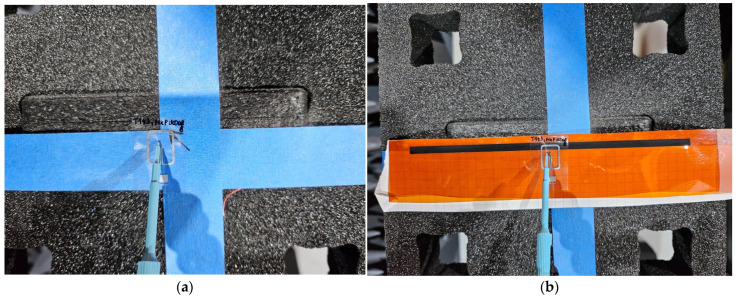
Measurements of TT-type microelectronic module: (**a**) without coupling; (**b**) with coupling to graphene antenna.

In the first step of the experimental research, the read range of the microelectronic modules, not yet coupled with the antenna modules, was measured (Figure 4a). Next, this parameter was determined for the complete RFID_LIG_ tags (Figure 4b), consisting of an antenna module inductively coupled with the graphene path at the center of the LIG sample. The obtained results were used to assess the impact of the attached graphene antenna on the transponder’s operational efficiency within the RFID system. The measurement results are summarized in Section 3.2.

## 3. Results

### 3.1. Technological Conditions of the LIG Sample Fabrication Process

**Figure 5:**
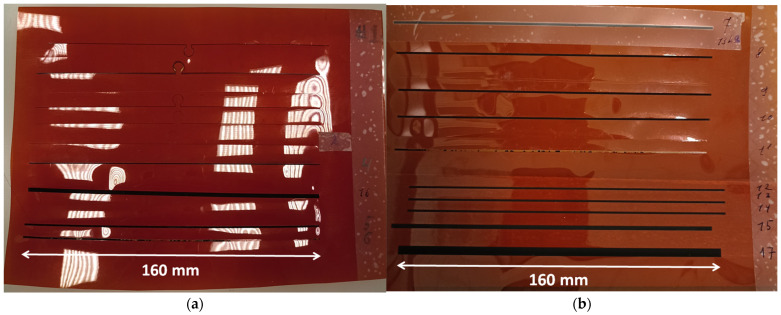
Test samples of graphene paths: (**a**) #1–#6, #16; (**b**) #7–#15, #17.

By adjusting the laser parameters and refining the method of fixing the Kapton substrate to the laser’s working table, seventeen test samples with conductive graphene paths were successfully prepared (Figure 5).

The main laser parameters adjusted during the research process, along with the resistance of the induced graphene paths, are presented in Table 2.

The samples #1 and #2 were designed in the shape of a dipole antenna with a small coupling loop in the middle. They measure 160 mm by 1 mm, as per the calculation provided in [13]. They were created using the “Cut CO_2_” laser mode, but regardless of the adjustments to the machine setup, the system automatically activated the two-way cutting process. This resulted in cutting through the Kapton substrate, leading to the absence of the desired conductive path. The simplification of the antenna shape solved this problem. The new design consisted of a filled rectangle with a length of 160 mm and was created in the “Engrave” mode. In samples #3 to #14, a filled rectangle with a path width of 1 mm was induced. The Kapton substrate was fixed to the hexagonal grid of the operating table using weights on the sides of the sheet, which helped counteract the thermal bending of the material during the engraving process. Further modifications to the laser parameters led to the creation of sample #5. The resistance of the induced graphene layer was 150 kΩ, which is too high a value for using it in the front-end circuits of UHF RFID transponders. In subsequent steps, the focus shifted to optimizing the technological process. In sample #6, the head distance from the substrate (OZ = 62.06 mm) was slightly adjusted, changing the focus of the light beam. As a result, a sample was obtained with graphene that was not bonded to the substrate and was removed during the ventilation of harmful residues from the engraving machine. In sample #7, the PPI parameter was slightly reduced, but this did not yield the expected results. Therefore, it was decided to set this parameter to a constant value of 1000 pulses per inch for the subsequent attempts. In sample #11, the laser power was reduced while its speed was increased. The effect of this modification was identical to that of sample #6. Overall, thanks to the adjustments made to the laser engraving settings, the resistance of the induced conductive path was reduced to 9.6 kΩ in sample #14.

For samples #15 and #16, the width of the designed rectangle was increased to 2 mm. In the case of #15 sample processing, the Kapton sheet was attached to a plexiglass plate using double-sided tape, while for sample #16, weights were used instead of adhesive tape. As a result of these improvements, a graphene path with a resistance of 4.77 kΩ @ #15 and 5.7 kΩ @ #16 was induced. Sample #17 was made identically to sample #15 (attaching Kapton to the plexiglass plate), but the width of the designed rectangle was increased to 4 mm. The graphene path obtained in this way had a resistance that was half the value of sample #15, measuring 2.29 kΩ.

**Figure 6:**
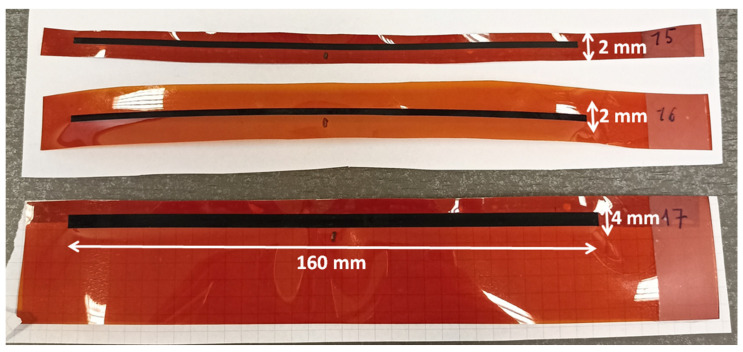
Samples of graphene paths #15–#17.

In the experiment, the OZ coordinate for the focused laser head was set to 54 mm. Samples #1 to #3, #6 and #12 to #17 were processed with a defocused laser beam, and graphene was induced only in the last group mentioned (OZ = 60.48 mm). Comparing samples #6 and #12, which had engraved at similar power and head speed parameters of laser device, it can be concluded that the quality of the graphene induction depends on the degree of laser defocusing. After analyzing the results, samples #15 to #17, which exhibited the lowest resistance in the antenna path, were selected for further investigation (Figure 6).

In order to estimate the stability of the graphene layers, the resistance of the paths for samples #15–#17 was measured, and the results are presented in Table 3. The first measurements were taken immediately after the graphene had been induced. During the measurements, brittleness of the created layers was observed. However, the tested paths were subjected to intense use. During the research, the unprotected graphene layer was repeatedly touched with fingers, measurement probes, and microelectronic modules. Also, during sample separation, the Kapton film was bent significantly. The samples were stored under room conditions but were frequently moved and used for other experiments not presented in the article. To observe the impact of environmental factors and mechanical stress on the graphene samples, the resistance of paths #15–#17 was measured again after 4.5 months from the first measurement (Table 3). Both measurement series were performed using the same method and equipment.

As can be observed, the resistance of all three samples increased, which would significantly impair the read range of the RFID_LIG_ tags in subsequent measurements. The degradation of the graphene paths was caused by the formation of microcracks in the graphene structure and the wear of the graphene layer during use. According to [36], changes in the graphene parameters may also be caused by exposure to natural environmental conditions (humidity, air temperature), which lead to the oxidation of both graphene and the substrate. Therefore, in future work, it would be worthwhile to focus on attempts to protect laser-induced graphene from long-term wear. This could involve covering the graphene with a protective coating as well as secondary graphene induction on a damaged surface as in [37].

### 3.2. Parameter Determination of RFID_LIG_ Transponders

The final stage of the investigations involved measuring the boundary parameters: forward *r_PwrMax_* and reverse *r_BtrMax_* operational read range, which define the interrogation zone of the tested RFID system. The *r_PwrMax_* defines the maximum range of the forward link. For RFID systems, it is determined based on the following relation:

(1) $$r_{PwrMax}=\frac{c}{4\pi f}\sqrt{\frac{P_{EIRPmax}}{P_{Pwr}}}$$

where *c* is the speed of light equal to 2.998 × 10^8^ m/s, *f* means the frequency, *P_EIRPmax_*—the maximum effective isotropic radiated power, and *P_Pwr_*—the minimum power required by the transponder for proper direct operation.

The *r_BtrMax_* defines the maximum range for reverse link. It is described by the following equation:

(2) $$r_{BtrMax}=\frac{c}{4\pi f}\sqrt{\frac{P_{Btr}}{P_{Rmin}}}$$

where *P_Btr_* represents the effective backscatter power utilized by the transponder during communication with the reader/writer device, and *P_Rmin_* means the sensitivity of the reader/writer device. A detailed mathematical analysis of these parameters is presented in [26].

**Figure 7:**
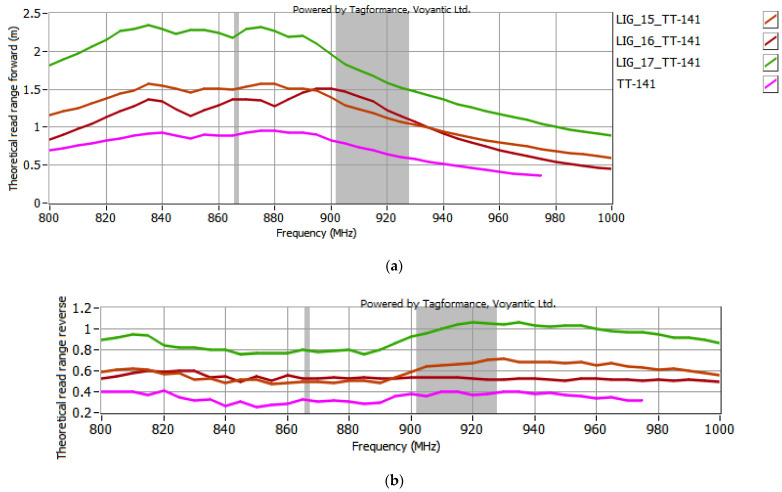
Read range of RFID_LIG_ transponders with TT-141: (**a**) forward (*r_PwrMax_*); (**b**) reverse (*r_BtrMax_*) link.

**Figure 8:**
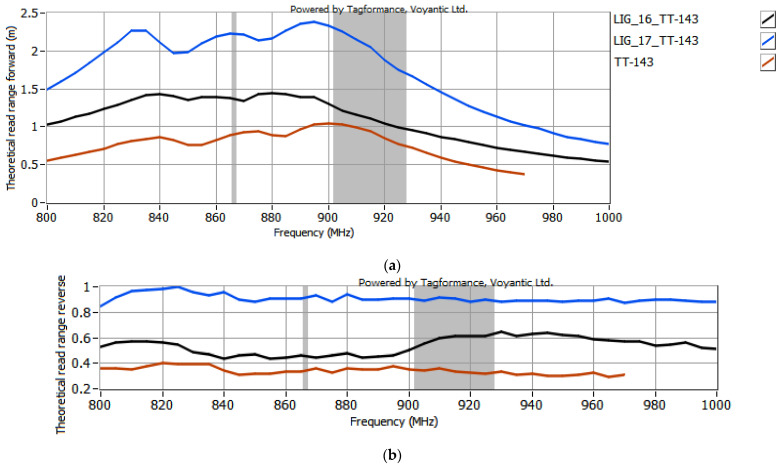
Read range of RFID_LIG_ transponders with TT-143: (**a**) forward (*r_PwrMax_*); (**b**) reverse (*r_BtrMax_*) link.

**Figure 9:**
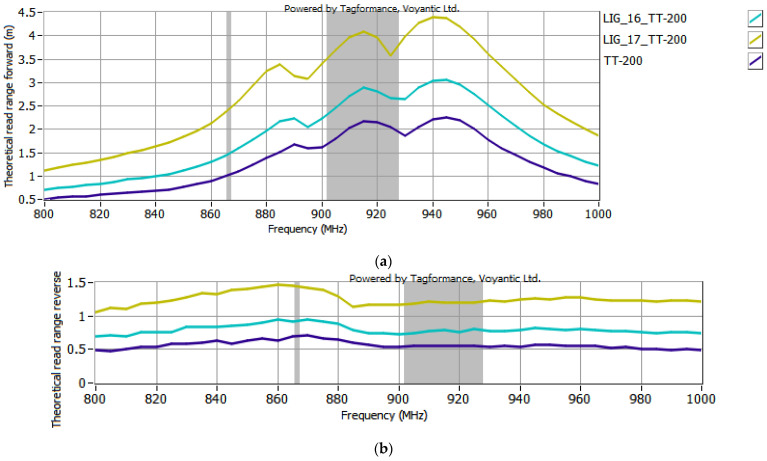
Read range of RFID_LIG_ transponders with TT-200: (**a**) forward (*r_PwrMax_*); (**b**) reverse (*r_BtrMax_*) link.

**Figure 10:**
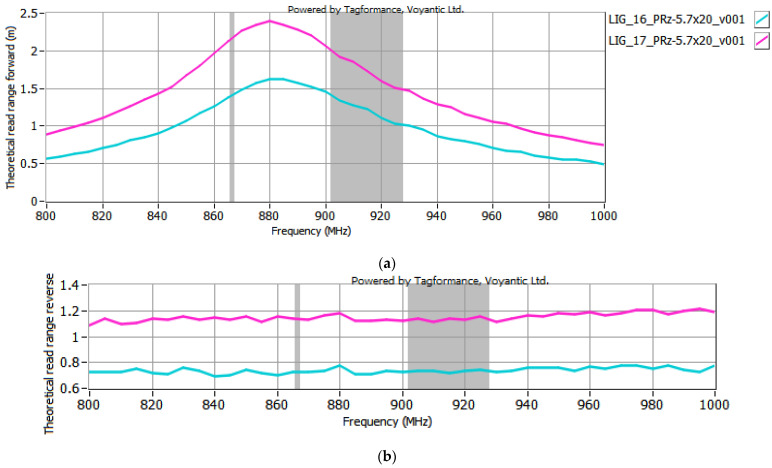
Read range of RFID_LIG_ transponders with 5.7x20_v001: (**a**) forward (*r_PwrMax_*); (**b**) reverse (*r_BtrMax_*) link.

The selected antenna modules with graphene paths, coupled with the microelectronic modules described in Section 2.2, were subjected to testing in the Tagformance System. According to the EPCglobal recommendations, the following receiver parameters were set in the Voyantic Tagformance Pro system: sensitivity of −70 dBm, antenna gain of 4 dBi, and the Wideband UHF Reference Tag V1 was used for calibrations. The configurations listed below were analyzed in the investigations:

- TT-141 module coupled with samples #15 to #17 (Figure 7);
- TT-143 module coupled with samples #16 and #17 (Figure 8);
- TT-200 module coupled with samples #16 and 17 (Figure 9);
- 5.7x20_v001 module coupled with samples #16 and #17 (Figure 10).

In the curve labels designated in Figure 7, Figure 8, Figure 9 and Figure 10, the notation LIG_xx represents the sample number of the antenna module, while TT-yyy refers to the name of the microelectronic module, derived from the Talkin’Things product name. The label 5.7x20_v001 in the last case indicates the microelectronic module that was specifically designed for the RFID_LIG_ conception. As mentioned in Section 2.2., the microelectronic module 5.7x20_v001 does not operate as a standalone transponder, so its read range could not be tested without being coupled with an antenna module. The charts in Figure 7, Figure 8, Figure 9 and Figure 10 also present the characteristics obtained for the TT-type microelectronic modules operating as the near-field tags, as suggested by the manufacturer.

After the first series of examinations (using the TT-141 microelectronic module, Figure 7), it was observed that the RFID_LIG_ tags exhibited very similar read range values when connected to antenna modules #15 and #16. In both cases, for the 900–930 MHz range, the *r_PwrMax_* of the tag ranged from 1 to 1.5 m, while the *r_BtrMax_* was around 0.6 m. Given the negligible difference in the resistance of these two graphene paths (only 1 kΩ), and considering the time-consuming nature of both conducting the measurements and processing the resulting data, continuing with both samples would offer little additional value while significantly prolonging the research. Therefore, to streamline the study and focus on the most promising configurations, it was decided to exclude sample #15 from further experiments.

Based on the results shown in Figure 7, Figure 8, Figure 9 and Figure 10, an increase in the read range for both forward and reverse links is observed when the microelectronic modules are coupled with the graphene paths. The use of graphene path #16 increases the read range in the forward direction (*r_PwrMax_*) of the TT-yyy microelectronic modules in the 900–930 MHz frequency band by about 0.75 m, 0.2 m, and 0.5 m compared to the TT-141, TT-143, and TT-200 near-field tags, respectively. The read range in the reverse direction also improves—the increase in *r_BtrMax_* relative to TT-141, TT-143, and TT-200 is approximately 0.15 m, 0.2 m, and 0.25 m in the same frequency range. A similar trend is observed when the TT-yyy microelectronic modules are coupled with the antenna module #17. The presence of the graphene path increases *r_PwrMax_* by approximately 1 m, 1 m, and 2 m, while *r_BtrMax_* improves by about 0.6 m, 0.5 m, and 0.75 m for TT-141, TT-143, and TT-200, respectively.

Regarding the 5.7x20_v001 microelectronic module, the read range in both the forward and reverse directions of the tag coupled with graphene path #17 is approximately 0.5 m and 0.4 m greater, respectively, compared to the tag coupled with graphene path #16. Although coupling enhances the read range in both directions, the increase in *r_BtrMax_* is less pronounced than the improvement in *r_PwrMax_*. Overall, the results indicate that the lower resistance of the LIG path leads to a greater read range for the evaluated RFID_LIG_ transponders.

**Figure 11:**
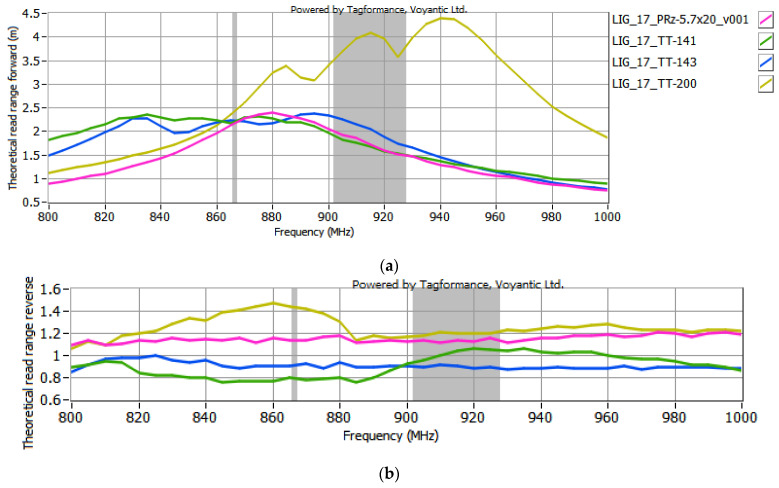
Read range of RFID_LIG_ transponders with sample #17 and all microelectronic modules: (**a**) forward (*r_PwrMax_*); (**b**) reverse (*r_BtrMax_*) link.

**Figure 12:**
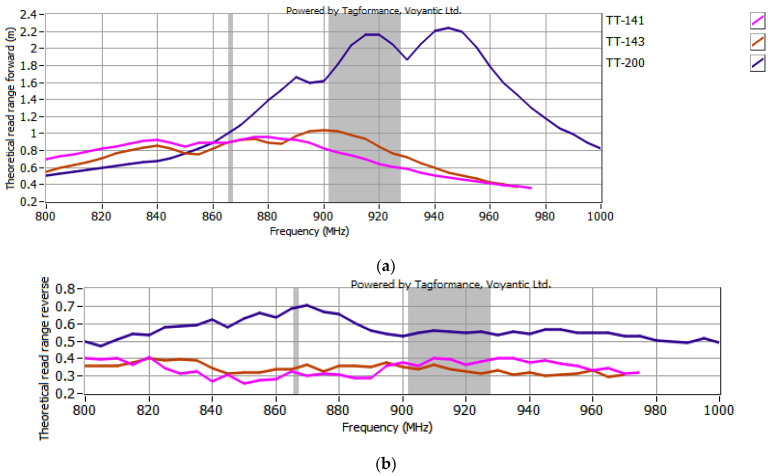
Read range of Talkin’Things microelectronic modules: (**a**) forward (*r_PwrMax_*); (**b**) reverse (*r_BtrMax_*) transmission.

The read range of RFID_LIG_ transponders composed of sample #17 coupled with all the used microelectronic modules is illustrated in Figure 11. To better evaluate the impact of the additional graphene path, the comparison of uncoupled microelectronic modules from Talkin’Things is presented in Figure 12.

Analyzing Figure 11 and Figure 12, it can be concluded that the highest read range is achieved when the TT-200 module is used. This parameter is generally smaller for other TT-yyy constructions, though the results are similar to each other. These findings align with expectations, as the transponder with the greatest read range consists of a TT-yyy module that inherently exhibits the best read range among the uncoupled modules, combined with a graphene path featuring the lowest resistance. Figure 11 and Figure 12 show identical relationships between the transponders, with the exception that adding a graphene path enhances the read range of all configurations. In this specific case, coupling with sample #17 doubled the measured values for the TT-yyy transponders.

The accuracy of the results obtained in Tagformance System may depend on several factors. Possible causes of measurement discrepancies could include the quality of inductive coupling that depend on the pressure applied to attach the microelectronic module to the antenna module; the curvature of the graphene paths resulting from LIG technology processing and substrate damage [38]; and the inaccurate placement of the microelectronic module relative to the antenna coupling circuit, exactly in the center of the conductive path, while the positioning may be disrupted when fixing pressure is applied. With a high level of experimental precision and accuracy as well as repeatability of the performed procedures, these discrepancies can be minimized, especially when comparative studies and numerical simulations are conducted.

## 4. Summary

The research presented in the paper is the first in a series dedicated to adapting the laser-induced graphene (LIG) method for manufacturing components of RFID_LIG_ transponders. The main objective was to identify the appropriate laser parameters for inducing graphene layers suitable for use in the UHF RFID tags.

In this study, two antenna shapes were considered: a dipole antenna with a loop coupling structure and a standard dipole antenna. The investigations confirmed that the laser system used is suitable for inducing conductive graphene paths with simple geometries. However, when inducing graphene in the form of a dipole antenna with a coupling loop, substrate damage occurred, leading to the exclusion of these samples from further research. For the standard dipole antenna, various laser parameters, substrate mounting methods, and graphene path widths were examined during the engraving process. The modification of these parameters significantly affects the conductivity of the obtained graphene layers. Notably, stiffening the Kapton substrate reduced the resistance of the graphene path under identical laser settings. Among the three mounting methods tested, the best results were achieved when the Kapton was attached to a rigid PVC surface using double-sided tape. This mounting approach minimized the deformation of the Kapton during the laser ablation.

The final parameters of the fabricated antenna module also depended on the designed size of the induced graphene layer. While the length of all produced samples was kept constant at 160 mm, three different widths were tested: 1 mm, 2 mm, and 4 mm. The study demonstrated that, under fixed laser parameters, increasing the width of the induced area reduces the resistance of the graphene sheets. Specifically, doubling the width resulted in a twofold decrease in resistance, while quadrupling the width led to a fourfold reduction.

Changes to the laser settings were primarily made in the “Engrave” mode because the samples were destroyed in the “Cut CO_2_” mode. The research indicates that the conductive graphene path is induced when three parameters: power, speed, and beam focusing are properly adjusted. The most optimal results were obtained with a defocused laser (OZ = 60.48 mm) and a setting for the ratio of %*P_max_* to %*v_max_* of 2:1.

The studies on the read range showed that coupling all the microelectronic modules with the selected graphene paths increased their operational read range. The greatest increase was achieved for graphene samples with lower resistance. Among the elaborated RFID_LIG_ devices, the best results were obtained when sample #17 was used, where coupling with the graphene path doubled the read range of the transponder.

The presented studies determine the qualitative impact of changes in laser settings on the results of the graphene induction process. Therefore, there is a need for in-depth research into the quantitative effect of specific laser parameters on the quality of the produced LIG layers, better tailored to the requirements of RFID transponders. Additionally, an open question remains regarding how to protect such delicate graphene paths from mechanical damage, for example, during wear or transport. In these studies, graphene was synthesized only on the Kapton substrate, so further research should also focus on other materials. This is especially relevant, as LIG technology can be applied in various industries, such as the textile, medical, food sector, etc. Conducting research in these directions could lead to further publications.

## Figures and Tables

**Table 1:** Parameter configuration of communication protocol ISO/IEC 18000-63.

Parameter	Value
Forward link	DSB-ASK, *T_ari_* =25 μs
Return link	*FM0*, 40 kHz
Command	Query

**Table 2:** Parameters of laser and measured resistance *R* of graphene paths.

Laser Mode	Sample Number	*p*, % of Max	*v*, % of Max	kHz_Cut_/ PPI_Engr_	OZ Coordinate	Path Width	*R*, kΩ
Cut CO_2_	1	7	5	5	62.06	1	*
	2	5	1	10	55.26	1	*
Engrave	3	5	1	1000	62.06	1	*
	4	5	6	1000	54	1	*
	5	9	5	1000	54	1	150
	6	9	5	1000	62.06	1	*
	7	10	5.2	750	54	1	13.35
	8	10	5.5	1000	54	1	13.47
	9	10	11	1000	54	1	12.8
	10	18	3	1000	54	1	16.3
	11	5	5.5	1000	54	1	*
	12	10	5.5	1000	60.48	1	11.5
	13	10.5	5.5	1000	60.48	1	10.4
	14	11	5.5	1000	60.48	1	9.6
	15	11	5.5	1000	60.48	2	4.77
	16	11	5.5	1000	60.48	2	5.7
	17	11	5.5	1000	60.48	4	2.29

* The sample was destroyed by the laser, resulting in an open circuit.

**Table 3:** Comparison of the resistance *R* of selected graphene samples measured after an interval of 4.5 months.

Date	*R_15_*, kΩ	*R_16_*, kΩ	*R_17_*, kΩ
4 October 2024	4.77	5.7	2.29
20 February2025	6	6.6	2.9

## Data Availability

All the calculated and measured data will be provided upon request to the correspondent authors via email with an appropriate justification.
